# Burnout and Self-Perceived Instructional Competence: An Exploratory Study of a Group of Italian Female Elementary School Teachers

**DOI:** 10.3390/ijerph17041356

**Published:** 2020-02-20

**Authors:** Monica Pellerone, Venerando Rapisarda, Maria Chiara Antonietta Trischitta, Ermanno Vitale, Tiziana Ramaci

**Affiliations:** 1 Faculty of Human and Social Sciences, University of Enna “Kore”, CAP 94100 Enna (EN), Italy; mariachiaraantonietta.trischitta@unikorestudent.it (M.C.A.T.); tiziana.ramaci@unikore.it (T.R.); 2 Occupational Medicine, Department of Clinical and Experimental Medicine, University of Catania, CAP 95131 Catania (CT), Italy; vrapisarda@unict.it

**Keywords:** burnout, stress, school teachers, mental health, instructional competence

## Abstract

Since the first half of the 1980s, burnout in teachers has been the object of particular attention by many international authors. Teachers are subject, more than other professions, to numerous and heavy pressures, covering the peculiarity of the profession. The objectives of the present research are to measure the predictive role of emotional exhaustion, depersonalization, and personal accomplishment on the quality of teaching in a group of elementary school teachers. We carried out a cross-sectional study involving 324 Sicilian female teachers, who worked in three school orders: Kindergarten, primary school, and the first year of middle school. Participants completed a socio-demographic questionnaire, the assessment teaching scale for primary school teachers (ECAD-EP), and the Maslach Burnout Inventory (MBI). In reference to the level of burnout, the correlation analysis underlined the presence of a positive correlation between: Emotional exhaustion and depersonalization; and a negative correlation between exhaustion and depersonalization. Furthermore, a predictive role of emotional exhaustion, depersonalization, and personal accomplishment on the quality of teaching in a group of primary school teachers was found.

## 1. Introduction

In Italy, an efficient school is linked to teachers’ effectiveness. This theme is constantly present in national and international debates and continues to stimulate discussion.

In the present study, we carried out an explanatory conceptual design of effective teaching behaviors and styles, which is linked to “new” variables [1,2], corresponding to the self-perceived skills of a group of teachers, who were affected by scholastic system collapse and by psychological illness (burnout).

The literature underlines that teachers at risk of burnout can, to a lesser extent than other teachers, put into practice in the classroom those behaviors and skills linked to “new” metacognitive variables, which allow the establishment of a functional organizational climate, and can represent a predictive character of professional performance and increased motivation of their students [1,2,3]. In contrast, teachers who possess the metacognitive attitude—considered as the ability to reflect on one’s cognitive activity in reference to a task—are able to manage their emotions when dealing with students, are assured when facing critical situations, and use different strategies in different classrooms [4].

From a psychological and social point of view, feelings of frustration and burnout represent some of the manifestations of uneasiness; these dynamics favor the crystallization of roles and dysfunctional behaviors towards the perception of metacognitive teaching processes.

Burnout has been recognized by the World Health Organization (WHO) [5] as a medical disorder, and a “problem associated with the profession”, in which the quality of life can be improved by maintaining meaningful personal relationships.

Burnout refers—according to the 11th Revision of the International Classification of Diseases (ICD11)—specifically to phenomena in the employment context and should not be applied to describe experiences in other areas of life. It is characterized by three dimensions: Feelings of energy depletion or exhaustion; increased mental distance from one’s job (or feelings of negativism or cynicism related to one’s job); and reduced professional efficacy [6]. This condition results from a particular form of professional discomfort, which is the outcome of chronic stress, an affects mostly people engaged in helping. The literature has shown that this disease is a phenomenon of international scope, which occurs frequently among teachers [7].

Although burnout has been primarily described as a condition that depends, first of all, on organizational factors, some studies have found that personality variables can be predisposing factors to the onset of the syndrome [8,9,10]. Individual factors include personality traits, career, previous experience, role, age, attitude, and didactic, emotional, and socio-relational skills [11]. Similarly, among the personality traits, there are the following dimensions: Anxiety; flexibility or inflexibility of the subject, which leads to stress and affects the variables related to the skills of interpersonal relationships and inner balance, which decreases the quality of the process of the interactive teaching process and learning; and finally, perception of the personal quality of life [12].

Within the context of quality of life and burnout, a person-oriented approach could firstly promote understanding of students, as unique individuals, by considering the many facets of quality of life experienced within a school environment, rather than considering each facet in isolation.

Thus, burnout negatively affects the ability to mediate and involve the class group (socio-emotional factor); the ability to manage social interactions (communicative-relational factor); and the capacity related to the teaching strategy (didactic factor).

Recent studies [7,13] have confirmed that teachers are subjected, more than other professions, to numerous and heavy pressures, related to the transformation of society towards a more multi-ethnic and multicultural lifestyle, as well as taking care of children with different types of disabilities, and the technological evolution.

Not all teachers are equally vulnerable to developing burnout, because the subjective efficacy and ability to make decisions represents protective factors for the manifestation of the syndrome [14]. It is worth considering a further aspect concerning the aging trend of school staff in Italy [15]. As the Organization for Economic Cooperation and Development (OECD) points out, the number of teachers over the age of 50 years in Italian schools has increased from 31.5% in 2002 to 48.68% in 2011 [15], making Italy the country with the highest share of teachers older than 50 years in the world [16]. The age of the working population has been attributed as a main emerging psychosocial risk, and these demographic changes pose urgent problems related to sustainability and active aging at work.

Literature related to the school environment has always investigated the relationship between the risk of teachers developing burnout syndrome and the possible effects on; protective factors [17,18,19,20,21]; the analysis of teaching contexts capable of promoting better working conditions [22]; the analysis of behavior with respect to evaluation standards [23]; and the analysis of decision styles [24].

Previous findings support the critical influence of a teacher’s well-being and psychophysical health on their performance [25,26,27,28,29]. A strong sense of well-being and psychophysical health in teachers promotes a firm commitment to the profession and control of metacognitive strategies, aimed at teaching students useful strategies to learn, remember, study, and self-evaluate their school performance [30,31]. Behaviors and teaching styles will be influenced by the state of health and predictors of the work and life quality of the teachers.

There is wide research indicating that, as teachers become more aware of their ability to teach, their self-efficacy increases. As a result, their self-competence improves by developing a metacognitive-self-regulatory approach through an inquiry process of reflective thinking on why, what, and how to teach [32,33].

As an extensive review of literature has clearly documented [34,35], teachers with a strong sense of efficacy exhibit greater levels of planning and organization [36], are more open to new ideas, and are more willing to experiment with new methods to better meet the needs of their students [15,37]. They also exhibit greater enthusiasm for teaching [36], have greater commitment to teaching, and are more likely to stay in teaching. In addition, they are likely to exert a determinant positive influence on students’ achievements and their own sense of efficacy [38,39].

Teachers with metacognitive attitudes, who have high levels of well-being or low burnout, are able to manage their emotions in relationships with students, feel more effective in facing critical situations, and use different strategies in different classrooms [40], such as functional coping strategies, defined as the efforts used to manage the internal or external demands that are appraised as potentially harmful and stressful to the individual [41,42].

Well-being, quality of life, and psychophysical health of teachers is an essential incentive to increase work performance, good learning processes, and the high motivation of students. Furthermore, these factors are strictly related to perceived self-efficacy in teaching, good performance, and control of teaching-metacognitive strategies. Other research findings suggest that well-being is a strong predictor of a teacher’s successful performance and metacognitive attitude in the classroom [43,44,45,46].

Although well-being and psychophysical health of teachers have important implications on the health and well-being of their recipients, studies dedicated to these workers and their consequences on the metacognitive style are quite rare, particularly in Italy [47,48,49].

Even though the studies cited above consider teaching-metacognitive strategies, in our study review, we investigate teaching-metacognitive strategies in direct correlation to burnout.

## 2. Research Objectives and Hypotheses

The literature distinguishes two types of dimensions of teachers’ competence: One that refers directly to the curricular sphere of their scientific path, and another which refers to the teachers’ qualities and the characteristics that define them, such as their personal, psychological, and technical instrumental qualities, as well as the interactive aspects [50,51,52]. These variables can modulate the teaching–learning process, giving rise to different teaching styles, and are made up of an integrated set of cognitive, affective, and social dimensions linked by a dynamic relationship [52,53].

The teaching styles adopted, in fact, somehow condition the different elements of teaching, because they interfere or alter the cognitive, affective, and social dimensions. Effective teachers therefore will most likely master different teaching styles, and having previously analyzed the situation, will know how to apply them [54].

For these reasons, within a metacognitive approach, this work examines the teaching variables inherent in the teacher as an intentional agent, who reflects and makes conscious decisions rather than being a mere translator of knowledge, taking into account the dual nature of their dynamic activity. These variables include; a more individual or introspective one (i.e., the emotional component), and a more social one, which can be further distinguished as a component of socio-relational and organizational adaptation.

The aim of this research is to measure the self-perceived teaching skills of a group of 324 teachers, of which 75.3% were curricular and 24.7% were specialist support teachers of the first education cycle of Sicilian schools, and measure the predictive role of the possible presence of emotional exhaustion, depersonalization, and job dissatisfaction on the quality of teaching into the both groups. This division on the basis of the type of role was made because the tasks required from both types of teachers are different: In particular, the support teachers guarantee continuous and stable support to students with disabilities; instead, the curricular teachers provide support to the all students in the classroom.

In particular, we hypothesized that:

- Years of experience, the number of students per class, and the presence of students with certified disability (students with intellectual or physical disability) influence the socio-emotional and communicative-relational ability of teachers; in the first research hypothesis, students with learning disabilities (LD) or ADHD were not included, because the literature examined underlines that the presence of students with special educational needs (i.e., LD or ADHD) does not seem to influence the relational and communication skills of the teacher;
- The personal accomplishment could be predictive of the emotional, socio-relational, and didactic abilities of teachers;
- The socio-emotional characteristics of teachers could be an important mediator of the relationship between the level of burnout and their competences and the level of burnout.

## 3. Methods

### 3.1. Participants and Procedure

The research involved 324 Sicilian teachers, aged between 20–66 years old (*M* = 45.78; *S.D*. = 10.16), of which 75.3% were curricular and 24.7% were specialist support teachers, who worked in elementary schools (kindergarten, primary school, and the first year of middle school).

Convenience sampling was used to recruit the participants; in particular, the participants were selected consecutively in order of appearance, according to their accessibility (also known as consecutive sampling). The sampling process was finished when the total number of participants (sample saturation) and/or the time limit (time saturation) were reached. This method resulted in the recruitment of only female teachers.

The research lasted for 1 year; administration of instruments took place during the school timetable. The consent of the school authorities and the teachers involved in the study was sought before the distribution and collection of the instruments. The questionnaires were anonymous, and the participants were informed of the aim and structure of the study. All participants provided written informed consent.

All procedures performed in the present study were in accordance with the 1964 Helsinki Declaration and its later and amendments or comparable ethical standards. The Internal Review Board (IRB) of the Faculty of Human and Social Sciences at the “Kore” University of Enna approved the present research.

### 3.2. Measures

Participants completed a socio-demographic questionnaire constructed ad hoc, the assessment teaching scale for primary school teachers (ECAD-EP), and the Maslach Burnout Inventory (MBI). Socio-demographic data were collected through the administration of a questionnaire constructed ad hoc and divided into three parts: The first to acquire basic information, such as age, sex, grade in which they teach; the second for establishing the number of students per class, presence of students with disabilities or specific learning disabilities; and the third for measuring the years of experience and the role at school (curricular or specialist support teacher).

The ECAD-EP questionnaire (or Escala de Evaluación de la Competencia Autopercibida of the Teacher of Primary Education) [51], created at the University of Valladolid, stems from the need to create a valid and accurate tool able to identify, and thus able to measure, the strategic competences of elementary school teachers. For research purposes, the Italian and integral version of the instrument was validated by Caggiano et al. [52]. This is a questionnaire on a Likert scale, consisting of 58 items grouped in three factor dimensions:

- Socio-emotional factor (Factor A), composed of variables related to interpersonal relationship skills and inner balance, which increase quality of the interactive teaching and learning process. They consist of the ability to mediate, to involve the class, and adaptability—the propensity to adequately deal with evolutionary tasks to adapt to unexpected requests due to changes in the external world—communicative sensitivity, the ability to establish a healthy cohabitation, affective involvement, empathy and self-efficacy;
- Communicative-relational factor (Factor B), including variables related to management of interactions and communicative dynamics. These involve the implementation of cognitive, metacognitive, and psycholinguistic skills, and socio-cultural and psycho-pedagogical abilities, which mediate the processes of teaching and learning. These variables correspond to assertiveness, affective, and executive leadership, conflict resolution, and non-verbal and para-verbal communication;
- Didactic factor (Factor C), which relates to the variables that refer to didactic skills of the development of actions, mainly related to the institutional role. It is linked to the management processes of teaching, aimed at achieving psychopedagogical results. It refers to skills of adaptation to new situations, planning, and didactic control [51,52].

Literature and the present study report good estimates of internal validity of the tool (Table 1).

The Maslach Burnout Inventory is a 22-item questionnaire, designed to assess the level of burnout of an individual [6]. It is a multidimensional questionnaire that addresses three areas of expertise: Emotional exhaustion (made up of 9 items), depersonalization (5 items), and personal accomplishment (8 items).

In particular, emotional exhaustion constitutes an answer related to a working situation that induces excessive emotional involvement and emotional overload; depersonalization manifests as a detached, sometimes decidedly negative and hostile attitude towards users; reduced personal accomplishment is substantiated in an exhausting feeling of inadequacy to establish an effective relationship of help with users and implies a reduced level of self-esteem and the attenuation of the desire for success. 

The Cronbach’s alpha coefficient, a measure of reliability or internal consistency, is equal to 0.87 for the emotional exhaustion scale, 0.71 for the depersonalization scale, and 0.78 for the personal accomplishment scale [55]. In the present study, the Cronbach’s alpha coefficient was equal to 0.85 for the emotional exhaustion scale, 0.67 for the depersonalization scale, and 0.71 for the personal accomplishment scale.

### 3.3. Data Analysis

All analyses were conducted with Statistical Package for the Social Sciences 26.0 (IBM Corporation, Armonk, NY, USA).

The descriptive analysis was used to assess the mean scores of all variables. In reference to the assessment teaching scale, the multivariate analysis of variance (MANOVA) was used to measure the influence of independent variables (age, years of experience, type of function, school grade, presence or absence of students with disabilities, and the number of students for class) on ECAD-EP sub dimensions.

In reference to the burnout level, the multivariate analysis of variance between-subjects design (MANOVA) was carried out to inspect the potential effect of the independent variables on MBI sub-dimensions.

To assess the relationships of the assessment teaching scale and burnout dimensions, a Pearson’s correlation coefficient was conducted.

Separate hierarchical regression analyses for each dependent variable were conducted to evaluate the contribution of the burnout on the strategic competences of teachers: In the first step, the demographic data were entered in the model, and in the second step, the MBI sub-dimensions were entered. A hierarchical regression is the general approach of estimating the regression equation by considering a defined set of variables. This analysis allows for the prediction or explanation of scores on a criterion variable on the basis of obtained scores on predictor variables, and provides knowledge of the relationships among all the variables A mediation analysis was conducted in order to identify whether the relationship between burnout levels and the teacher’s social-relational skills and the relationship between burnout and teachers’ didactic skills were mediated by their skills to manage and manifest emotional abilities. A mediation analysis was used since the independent variables were theoretically organized into exogenous causes and endogenous causes: Within our theoretical framework, the exogenous causes were represented by dimensions of the burnout (i.e., the explanatory variables for which the variability was given and not explained by the model); the endogenous causes were represented by emotional capacities of the teachers (i.e., those explanatory variables for which the variability was partially explained by the model).

## 4. Results

### 4.1. Preliminary Analyses

A descriptive analysis was conducted in order to investigate the socio-demographic data. In reference to the school grade: 70.1% of participants worked in a primary school, 16.7% in first year of middle school, followed by 13.3% in kindergarten. The participating teachers in this research had an average experience of 19.60 years (with a range between 1–42 years and standard deviation equal to 11.49). In reference to their role at school, 75.3% were curricular teachers, and 24.7% were specialist support teachers.

The average number of students for each classroom was equal to 21.42 (with a range between 6–30 students).

In reference to the presence or absence of students with difficulties: 51.9% declared to have students with specific learning disabilities (SLD) or Attention Deficit Hyperactivity Disorder (ADHD), followed by 23.8% who declared to have students with certified intellectual or physical disabilities, and 24.4% who declared to not have any students with difficulties or disabilities in the classroom.

A descriptive analysis was conducted in order to investigate the average scores obtained by the participants in the administration of ECAD-EP (Table 2).

A descriptive analysis was conducted in order to investigate the average scores obtained by the participants in the administration of MBI (Table 3); the same analysis was carried out in order to quantify the level of burnout, divided across low, medium, and high profiles (Table 4).

In reference to assessment teaching, Table 5 underlines the presence of correlations between subdimensions.

In reference to the level of burnout, the correlation analysis underlines the presence of a positive correlation between: Emotional exhaustion and depersonalization (r = 0.29; *p* < 0.001) and a negative correlation between exhaustion and depersonalization (r = −0.11; *p* < 0.05).

### 4.2. Results

In reference to the ECAD-EP scores, the first MANOVA was carried out in order to verify the influence of personal variables (age, years of experience, and role) on the socio-emotional, communicative-relational, and didactic factors.

Regarding to the socio-emotional factor, the MANOVA shows how age seems to influence the cohabitation ability (F = 1.62; *p* < 0.05); furthermore, years of experience seem to influence cohabitation (F = 1.59; *p* < 0.05), and above all, mediation (F = 1.99; *p* < 0.01), and self-efficacy (F = 1.88; *p* < 0.01). The Tukey’s post hoc test shows that that older teachers have a higher cohabitation ability than younger teachers; and teachers with more experience seem to show a higher level of cohabitation, mediation, and self-efficacy than those with less experience.

Furthermore, the results of the present analysis underline that the interaction between age and years of experience seems to influence cohabitation (F = 1.58; *p* < 0.05); moreover, interaction between age, experience, and position seems to influence cohabitation (F = 2.30; *p* < 0.05), affective involvement (F = 2.45; *p* < 0.05), and group dynamic ability (F = 2.09; *p* < 0.05). The descriptive analyses underline that older teachers with more experience tend to manifest elevated cohabitation ability; furthermore, older curricular teachers with more experience present a higher ability of cohabitation, affective involvement with their students, and capacity to cope with group dynamics.

In reference to the ECAD-EP scores, another MANOVA was carried out in order to verify the influence of organizational dimensions (number of students, the presence or absence of students with disabilities, grade in which teaching) on the socio-emotional, communicative-relational, and didactic factors.

In reference to the emotional factor, the data analysis underlines how the presence of disabled students (F = 4.00; *p* < 0.05), and the interaction of disabled students (with certified physical or intellectual disability) and total students per class (F = 2.29; *p* < 0.01), tend to influence the group dynamics. The descriptive analyses underline that numerous classes with disabled students present a reduced level of functional group dynamics.

In reference to the communicative-relational factor, non-verbal communication (F = 3.23, *p* < 0.05), conflict resolution ability (F = 3.00, *p* < 0.05), and above all, effective leadership (F = 5.85, *p* < 0.01) seem to be influenced by the grade in which teachers work. The Tukey’s post hoc underlines that teachers working at a lower level of education seem to show a higher level of non-verbal communication and conflict resolution capacity, but teachers working at a higher level of education tend to manifest a lower level of effective leadership.

Furthermore, MANOVA underlines how the presence of disabled students influences the use of executive leadership (F = 5.61; *p* < 0.05); and the number of students influences the use of pre-verbal communication (F = 1.90; *p* < 0.05). The descriptive analyses underlines that in a group context with disabled students, teachers tend to use a higher level of executive leadership; and in classes with fewer students, teachers tend to use a reduced level of pre-verbal communication.

Regarding the didactic factor, the grade only seems to influence the perception of institutional control (F = 4.26, *p* < 0.05), which appears more elevated in the superior grade (as underlined by the Tukey’s post hoc analysis).

The MANOVA, carried out to verify the influence of independent variables on burnout subdimensions, underlines that the years of teaching experience influences emotional exhaustion (F = 1.11; *p* < 0.05) and general level of burnout (F = 2.77; *p* < 0.01), which is also influenced by the number of students per class (F = 2.03; *p* < 0.05) and by the type of role (F = 5.73; *p* < 0.05). The Tukey’s post hoc underlines that: Teachers with more experience manifest reduced levels of emotional exhaustion and general burnout; specialist support teachers and curricular teachers who work in numerous classes tend to manifest higher levels of burnout.

Furthermore, the MANOVA emphasizes the influence of an interaction effect between experience and grade in which they teach on personal accomplishment (F = 2.28; *p* < 0.05), and above all, on the total level burn out (F = 3.02; *p* < 0.05). In particular, the descriptive analysis shows that participants with more experience, who teach in lower grade schools, tend to manifest an elevated personal accomplishment, but reduced general burnout.

Moreover, the interaction between experience and the number of students seems to influence the level of emotional exhaustion (F = 1.17; *p* < 0.05), depersonalization (F = 2.23; *p* < 0.01), and general level of burnout (F = 2.37; *p* < 0.01); the interaction between experience and role seems to influence the level of burnout (F = 3.94; *p* < 0.01), which appears also to be influenced by the interaction between the grade in which they teach and number of students per class (F = 9.31; *p* < 0.001). In particular, descriptive analyses show that teachers with reduced years of experience, who work in numerous classes, manifest higher emotional exhaustion, depersonalization, and general burnout. Furthermore, teachers with a lower level of burnout are the curricular teachers with more experience; as opposed to teachers with higher total levels of burnout, who seem to be those who work in numerous classes at lower grade schools.

Multiple regression analyses were conducted to evaluate the contribution of the burnout sub-dimensions on the teachers’ strategic skills. The first regression analysis underlines that predictors of the socio-emotional factors are (Table 6): In the first step, only the years of experience (β = 0.29) explaining 9.3% of the total variance; in the second step, the level of personal accomplishment (β = 0.71) explaining 36% of the total variance.

Similarly, the second regression analysis underlines that predictive factors of the communicative relational dimension are (Table 7): In the first step, the years of experience (β = 0.27) explaining 8% of the total variance; and in the second step, the level of personal accomplishment (β = 0.51) explaining 28% of the total variance.

The third regression analysis underlines that predictor factors of the didactic dimension are (Table 8): In the first step, the years of experience (β = 0.19) and the number of students (β = −0.18) explaining 15% of the total variance; in the second step, the level of personal accomplishment (β = 0.52) explaining 44% of the total variance.

In order to verify whether reduced emotional capacities mediate the effect of burnout on teachers’ social and didactic skills, mediation analyses were conducted. The first linear regression analysis shows the presence of a simple relationship between the emotional exhaustion and the didactic skills of the teacher (β = −0.13, *p* < 0.05), as further confirmed by the Univariate Analysis of Variance [F_(1,323)_ = 50.66, *p* < 0.05]. Upon adding the hypothesized mediator, or the Factor A, into the regression model, the beta value of the emotional exhaustion becomes insignificant (β = 0.03, *p* > 0.05), while the beta value of the factor hypothesized as a mediator is highly significant (β = 0.77, *p* <0.001); therefore, it is possible to confirm that the socio-emotional characteristics (Factor A) are an important mediator of the relationship between burnout and Factor C (Table 9).

Another linear regression analysis shows the presence of a simple relationship between the emotional exhaustion and the socio-relational skills of the teacher (β = −0.16, *p* < 0.01), as further confirmed by the Univariate Analysis of Variance [F_(1,323)_ = 80.39, *p* < 0.01]. Adding the hypothesized mediator, or the Factor A, into the regression analysis, the beta value of the emotional exhaustion becomes insignificant (β = −0.007, *p* > 0.05), while the beta value of the factor hypothesized as mediator is highly significant (β = 0.73, *p* < 0.001), therefore data confirm that the socio-emotional characteristics (or Factor A) are a mediator of the relationship between level of Burnout and Factor B (Table 10).

## 5. Discussion

The objectives of the present research were to measure the predictive role of emotional exhaustion, depersonalization, and personal accomplishment on the quality of teaching in a group of female elementary school teachers, divided into support teachers—whose role is to guarantee continuous and stable support to students with physical or intellectual disabilities—and curricular teachers, whose role is to provide support to all the students.

The first research hypothesis was confirmed, because results show that, in reference to the socio-emotional ability, teachers working at a higher level of education with fewer years of experience seem to show reduced levels of empathy; furthermore, numerous classes with intellectually or physically disabled students present a reduced level of functional group dynamics.

In reference to the communicative-relational ability, the data underline that teachers working at a lower level of education seem to show a higher level of non-verbal communication and conflict resolution capacity, whereas teachers working at a higher level of education tend to manifest a lower level of effective leadership. Moreover, in a group context with disabled students, teachers tend to use a higher level of executive leadership, and in classes with fewer students, teachers tend to use a reduced level of pre-verbal communication. Regarding the didactic capacity, the grade seems to influence the perception of institutional control, which appears more elevated in the superior grade.

The present data confirm a study of Serrano and Pons [56], which underlines that all the classifications of the teaching-learning processes share some main aspects: They fulfill a social and socializing function [57]; they have an affective component (attribution of meaning) and a cognitive component (construction of meaning); and they are mediated processes. Furthermore, literature underlines that teachers, during their professional path, discover that their good academic preparation is insufficient, because other behaviour problems, attitudes, and relationships overwhelm their plans. Social, affective, and emotional problems are mixed up in the schools and classrooms, and teachers are not always sufficiently prepared to face and solve them [58,59].

For this reason, it is considered important to pay attention to the psychological aspects that mediate and modulate the teaching-learning processes, dynamizing entities that lend value, and human meaning to the instructive activities.

The second research hypothesis, related to the predictive role of personal accomplishment on the emotional, socio-relational, and didactic abilities of teachers, was partially confirmed: Only more experience and a higher level of personal accomplishment seem to be predictive variables of the emotional, socio-relational, and didactic abilities of teachers. This data is confirmed by the literature, which states that greater personal satisfaction determines the presence of elevated socio-relational resources of the teacher. These resources are useful in the case of difficult interactions with parents; and also when there are difficulties with their teaching peers and the school’s administration [60,61,62].

Furthermore, confirming the literature, teachers with more years of experience display a greater ability to deal with classroom management demands, difficulties with the emotional climate within the school, difficulties regarding the teacher–student interactions, and the imbalance between these demands and the available resources [63].

According to these latest results, it is possible to confirm that burnout has become a significant part of the teaching climate because it can affect both the personal stability of individual teachers, as well as the teaching field at large.

Moreover, the socio-emotional characteristics of teachers seem to be an important mediator of the relationship between their emotional exhaustion and didactic strategies; similarly, the socio-emotional characteristics of teachers seem to be an important mediator of the relationship between their emotional exhaustion and the socio-relational competences.

The data appear to confirm the findings of Montalbetti [64], which underlines that teachers educate on the basis of their degree of emotional maturity and self-control, or in relation to their level of awareness of their affective dimension.

The empathic predisposition of the teacher, and above all, the ability to recognize personal emotions [65] favors the process of understanding others by reading how their experience had consequences for them.

In fact, today’s school system is called upon to undertake a new educational enterprise, which is to transform emotions and sociality into transversal skills which are no longer considered occasional or intrusions, nor not very pertinent compared to traditional knowledge [66].

Nowadays, students play an active role in the learning process; they are engaged and interested in the construction of meanings, and work with their peers for the construction of knowledge [67,68]. In contrast, teachers are committed in their role as mediators to offer learning opportunities, facilitated by an inclusive and motivating environment.

This causes a change of perspective and study of the teaching-learning processes, from a cognitive model to a constructivist one [69]. In fact, in the past the research trends in educational psychology have been directed to the cognitive activities underlying the actions of the teachers [70]; today, the variables studied in the literature consist of an integrated system of cognitive, affective, social, and behavioral dimensions [71,72].

The teaching-learning processes are influenced by all of these variables, which are dynamic forces of change, expressed in terms of motivated teaching skills repertoires [50]; as such, teaching styles represent a defining variable specification of the teaching process. The flexible and versatile use of teaching styles is essential to address different ways of learning, and to consolidate what has been learned by students. Although pupils have different cognitive styles, such as methods of approach to the task, capacity for abstraction, types of thought and intelligence, it is not possible to put into practice strictly individualized strategies [73]; changing teaching techniques instead, one can experience the individual differences, which are largely grouped into major categories [74].

Results of the present research underline that it is possible, therefore, to think about the style of teaching as the result of choices that teachers work by in different various dimensions of their professionalism: Communicative, cognitive, relational, and evaluative.

Information obtained by the teachers interviewed allowed us to identify critical issues of working in different school contexts, and give them an order of priority, according to which actions for improvement could be planned.

In particular, according to the present research, among the major critical issues are: Physical and emotional fatigue; the presence of aloof and apathetic students, colleagues and interpersonal relationships; and feelings of frustration due to the non-implementation of their expectations [75,76,77]. Furthermore, the difficulties of young Sicilian teachers to securely enter a profession is a risk factor for establishing a personal and social identity, as well as for their mental health [78,79,80]; they may even impact one’s awareness, especially because the principle on which postmodern society is based is the demonstration of the value of one’s own resources [81].

The study presents some limitations. A limitation of the study is the cross-sectional measurement. It was not possible to test the causal relationships proposed in the theoretical framework. A further limitation of the study was the use of convenience sampling methods for data collection. While cross-sectional convenience samples may prove useful in exploring theoretical models, such as the one identified in the present study, caution should be exercised while generalizing the results beyond the current research. Furthermore, the teachers’ ability and burnout levels were measured through the use of self-reporting, which poses a risk of misleading information or social desirability. This study could lead to inherent bias because of unmeasured confounders, including workloads and resources for teachers, genetic risk for depression, past psychiatric illness, and substance abuse, which should be measured in burnout research [67]. Finally, results should be interpreted with caution due to the participants involved, in particular, because there were no male teachers in our study, which makes generalizations difficult.

## 6. Conclusions

Relational and communicative competence, the assertive competence of the teacher, good emotional skills, the willingness to listen to oneself and to the student, attitudes of understanding, acceptance, and participation are factors influencing teaching quality.

The ability to recognize and regulate emotions and empathy in a functional way, good convictions of perceived personal efficacy, and the tendency to seek profitable solutions to problems stem the opportunities for conflict and increase the subjective well-being of teachers, improving the pedagogical relationship and the quality of learning.

A solid belief in the adequacy of one’s emotional and relational skills determines the effort and commitment of individuals to build authentic interpersonal relationships.

Collaborating, working in a team, and sharing repertoires and meanings already in the training phase of the profession corroborate the advancement of management skills relating to the professional role of the teacher and better educational effectiveness.

Transposing these positions into practice becomes an essential requirement if the skills mentioned above are recognized as essential and necessary components of teaching professionalism, if we take a vision of the school as a welcoming and educating community, and if we support the role of the teacher as a professional, which tends to the development and enhancement of all aspects of the person, considered in its entirety and globality.

## Figures and Tables

**Table 1:** The estimates of internal validity of the assessment teaching scale for primary school teachers (ECAD-EP).

Variables	Cronbach’s Alpha	Cronbach’s Alpha in the Study
Cohabitation	0.78	0.72
Empathy	0.74	0.69
Communicative Adaptation	0.63	0.55
Communicative Sensitivity	0.62	0.72
Mediation	0.56	0.75
Emotional Involvement	0.54	0.39
Group Dynamics	0.53	0.73
Self-Efficacy	0.51	0.66
Non-verbal communication	0.76	0.67
Assertiveness	0.69	0.55
Executive Leadership	0.62	0.52
Conflict Resolution	0.55	0.63
Paraverbal Communication	0.46	0.35
Effective Leadership	0.46	0.38
Institutional control	0.80	0.72
Planning	0.71	0.65
Adaptation to New Situations	0.55	0.69

**Table 2:** Descriptive analysis in reference to the ECAD-EP instrument.

Factors	Variables	N	Min.	Max	M.	S.D.
**Socio-emotional factor**	Cohabitation	324	2.57	5.00	4.020	0.472
	Empathy	324	2.50	5.00	4.395	0.597
	Communicative Adaptation	324	3.00	5.00	4.521	0.464
	Communicative Sensitivity	324	1.50	5.00	4.790	0.413
	Mediation	324	2.80	5.00	4.455	0.474
	Emotional Involvement	324	1.50	5.00	4.184	0.651
	Group Dynamics	324	2.57	5.00	4.215	0.511
	Self- Efficacy	324	3.00	5.00	4.488	0.414
**Communicative-relational factor**	Non-verbal communication	324	2.33	5.00	4.093	0.611
	Assertiveness	324	2.50	5.00	4.313	0.613
	Executive Leadership	324	2.50	5.00	4.477	0.548
	Conflict Resolution	324	3.00	5.00	4.469	0.498
	Paraverbal Communication	324	2.00	5.00	4.082	0.682
	Effective Leadership	324	2.00	5.00	4.216	0.671
**Didactic factor**	Institutional control	324	2.33	5.00	4.530	0.546
	Planning	324	2.50	5.00	4.495	0.455
	Adaptation to New Situations	324	2.00	5.00	4.392	0.626

**Table 3:** Descriptive analysis in reference to the MBI instrument.

Variables	N	Min.	Max.	M.	S.D.
Emotional Exhaustion	324	0.75	5.75	2.348	1.121
Depersonalization	324	0.00	4.00	1.431	0.712
Personal Accomplishment	324	0.75	5.63	4.48	0.815
Total level of Burnout	324	0.91	4.68	2.640	0.567

**Table 4:** Descriptive analysis in reference to the level of burnout.

Level	Emotional Exhaustion		Depersonalization		Personal Accomplishment	
	N	Percentage	N	Percentage	N	Percentage
Low	169	45.2	346	92.5	207	55.3
Medium	132	35.3	18	4.8	93	24.9
High	73	19.5	10	3.7	74	19.8
Total	374	100.0	374	100.0	374	100.0

**Table 5:** Correlation analysis between ECAD-EP subdimensions.

Variables	a.	b.	c.	d.	e.	f.	g.	h.	i.	l.	m.	n.	o.	p.	q.	r.
Cohabitation	1															
Empathy	0.440 **	1														
Com. Adaptation	0.462 **	0.431 **	1													
Com. Sensitivity	0.280 **	0.258 **	0.288 **	1												
Mediation	0.513 **	0.392 **	0.522 **	0.293 **	1											
Emotional Involv.	0.433 **	0.211 **	0.442 **	0.211 **	0.428 **	1										
Group Dynamics	0.493 **	0.392 **	0.506 **	0.299 **	0.595 **	0.408 **	1									
Self-Efficacy	0.490 **	0.406 **	0.481 **	0.340 **	0.631 **	0.488 **	0.620 **	1								
Non-verbal com.	0.418 **	0.286 **	0.272 **	0.327 **	0.508 **	0.326 **	0.525 **	0.621 **	1							
Assertiveness	0.433 **	0.268 **	0.305 **	0.213 **	0.446 **	0.288 **	0.424 **	0.435 **	0.417 **	1						
Executive Lead.	0.567 **	0.284 **	0.388 **	0.282 **	0.577 **	0.379 **	0.467 **	0.540 **	0.519 **	0.588 **	1					
Conflict Resol.	0.523 **	0.358 **	0.394 **	0.366 **	0.538 **	0.418 **	0.570 **	0.658 **	0.587 **	0.460 **	0.625 **	1				
Paraverbal Com.	0.232 **	0.272 **	0.216 **	0.155 **	0.228 **	0.142 **	0.378 **	0.372 **	0.241 **	0.255 **	0.226 **	0.345 **	1			
Effective Lead.	0.364 **	0.285 **	0.288 **	0.199 **	0.375 **	0.196 **	0.419 **	0.457 **	0.371 **	0.315 **	0.338 **	0.416 **	0.482 **	1		
Inst. Control	0.499 **	0.363 **	0.522 **	0.326 **	0.673 **	0.409 **	0.581 **	0.613 **	0.547 **	0.440 **	0.567 **	0.567 **	0.330 **	0.418 **	1	
Planning	0.508 **	0.381 **	0.442 **	0.321 **	0.516 **	0.380 **	0.550 **	0.609 **	0.507 **	0.433 **	0.527 **	0.602 **	0.270 **	0.390 **	0.574 **	1
Adaptation	0.434 **	0.305 **	0.506 **	0.294 **	0.498 **	0.375 **	0.458 **	0.508 **	0.404 **	0.423 **	0.421 **	0.449 **	0.220 **	0.303 **	0.488 **	0.523 *

**Notes:** ** *p* < 0.01, two-tailed; * *p* < 0.05, two-tailed.

**Table 6:** Multivariate two-steps hierarchical modeling of burnout on the socio-emotional factor (Factor A).

Model	Variables	B	S.E.	Beta	t	P
1	Constant	4.545	0.164		27.755	0.000
	Age	−6.407	0.000	−0.020	−0.363	0.717
	Years of experience	0.009	0.002	0.286	5.257	0.000
	Grade	−0.062	0.036	−0.097	−1.705	0.089
	Role	−0.014	0.044	−0.017	−0.314	0.753
	Number of students	−0.006	0.005	−0.061	−1.143	0.254
	Disabled students	−0.024	0.047	−0.029	−0.511	0.609
2	Constant	3.811	0.172		22.169	0.000
	Age	0.000	0.000	−0.043	−0.941	0.347
	Years of experience	0.007	0.001	0.230	4.900	0.000
	Grade	0.011	0.032	0.017	0.336	0.737
	Role	−0.030	0.037	−0.038	−0.813	0.417
	Number of students	−0.008	0.004	−0.077	−1.694	0.091
	Disabled students	−0.022	0.040	−0.027	−0.560	0.576
	Emotional exhaustion	0.096	0.081	0.309	1.189	0.236
	Depersonalization	−0.014	0.049	−0.028	−0.278	0.781
	Personal accomplishment	0.306	0.062	0.713	4.911	0.000
	Total level burnout	−0.339	0.198	−0.549	−1.710	0.088

**Abbreviations**: SE, standard error; β, beta standardized coefficients.

**Table 7:** Multivariate two-steps hierarchical modeling of burnout on the communicative-relational factor (Factor B).

Model	Variables	B	S.E.	Beta	T	P
1	Constant	4.420	0.202		21.872	0.000
	Age	−5.219	0.000	−0.013	−0.240	0.811
	Years of experience	0.010	0.002	0.269	4.898	0.000
	Grade	−0.053	0.045	−0.068	−1.178	0.240
	Role	−0.024	0.054	−0.024	−0.446	0.656
	Number of students	−0.005	0.007	−0.038	−0.693	0.489
	Disabled students	−0.052	0.057	−0.052	−0.906	0.366
2	Constant	3.503	0.222		15.767	0.000
	Age	0.000	0.000	−0.031	−0.644	0.520
	Years of experience	0.008	0.002	0.222	4.485	0.000
	Grade	0.018	0.041	0.023	0.434	0.665
	Role	−0.042	0.048	−0.042	−0.864	0.388
	Number of students	−0.006	0.006	−0.047	−0.972	0.332
	Disabled students	−0.049	0.051	−0.049	−0.949	0.344
	Emotional exhaustion	0.007	0.105	0.019	0.069	0.945
	Depersonalization	−0.038	0.064	−0.063	−0.596	0.552
	Personal accomplishment	0.266	0.080	0.506	3.302	0.001
	Total level burnout	−0.111	0.256	−0.147	−0.434	0.664

**Abbreviations**: SE, standard error; β, beta standardized coefficients.

**Table 8:** Multivariate two-steps hierarchical modeling of burnout on the didactic factor (Factor C).

Model	Variables	B	S.E.	Beta	T	P
1	Constant	4.543	0.215		21.129	0.000
	Age	0.000	0.000	−0.054	−0.976	0.330
	Years of experience	0.007	0.002	0.190	3.417	0.001
	Grade	−0.071	0.048	−0.086	−1.481	0.140
	Role	0.060	0.058	0.057	1.033	0.303
	Number of students	−0.010	0.007	−0.175	−2.367	0.043
	Disabled students	0.022	0.061	0.021	0.364	0.716
2	Constant	3.649	0.241		15.163	0.000
	Age	0.000	0.000	−0.071	−10.410	0.160
	Years of experience	0.006	0.002	0.142	20.768	0.006
	Grade	0.005	0.044	0.006	0.108	0.914
	Role	0.043	0.052	0.041	0.819	0.413
	Number of students	−0.011	0.006	−0.108	−2.710	0.038
	Disabled students	0.023	0.055	0.022	0.413	0.680
	Emotional exhaustion	0.047	0.113	0.117	0.415	0.678
	Depersonalization	−0.064	0.069	−0.101	−0.921	0.358
	Personal accomplishment	0.284	0.087	0.516	3.264	0.001
	Total level burnout	−0.173	0.277	−0.219	−0.625	0.532

**Abbreviations**: SE, standard error; β, beta standardized coefficients.

**Table 9:** Regression analysis: Factor A as mediator of the relationship between burnout and Factor C.

Model	Variables	B	S.E.	Beta	T	P
1	Constant	0.082	0.217		0.379	0.705
	Emotional exhaustion	0.012	0.015	0.031	0.851	0.396
	Factor A	0.993	0.047	0.773	21.109	0.000

**Abbreviations**: SE, standard error; β, beta standardized coefficients.

**Table 10:** Regression analysis: Factor A as mediator of the relationship between Burnout and Factor B.

Model	Variables	B	S.E.	Beta	T	P
1	Constant	0.390	0.222		1.757	0.080
	Emotional exhaustion	−0.003	0.015	−0.007	−0.180	0.857
	Factor A	0.887	0.048	0.724	18.434	0.000

**Abbreviations**: SE, standard error; β, beta standardized coefficients.
